# Analysis of common targets for circular RNAs

**DOI:** 10.1186/s12859-019-2966-3

**Published:** 2019-07-02

**Authors:** Ya-Chi Lin, Yueh-Chun Lee, Kai-Li Chang, Kuei-Yang Hsiao

**Affiliations:** 1 Department of Plant Pathology, College of Agriculture and Natural Resources, National Chung Hsing University, Taichung, 40227 Taiwan; 2 Department of Biotechnology, Asia University, Taichung, 41354 Taiwan; 3 Department of Radiation Oncology, Chung Shan Medical University Hospital, Taichung, 40201 Taiwan; 4 School of Medicine, Chung Shan Medical University, Taichung, 40201 Taiwan; 5 Department of Physiology, National Cheng Kung University, Tainan, 70101 Taiwan; 6 Institute of Biochemistry, College of Life Sciences, National Chung Hsing University, Taichung, 40227 Taiwan; 7 Program in Translational Medicine, College of Life Sciences, National Chung Hsing University, Taichung, 40227 Taiwan; 8 Rong Hsing Research Center for Translational Medicine, College of Life Sciences, National Chung Hsing University, Taichung, 40227 Taiwan; 9 Bachelor Program of Biotechnology, College of Agriculture and Natural Resources, National Chung Hsing University, Taichung, 40227 Taiwan

**Keywords:** Circular RNA, microRNA, Common targets, miRNA sponge

## Abstract

**Background:**

The studies of functions of circular RNAs (circRNAs) are heavily focused on the regulation of gene expression through interactions with multiple miRNAs. However, the number of predicted target genes is typically overwhelming due to the synergistic complexity caused by two factors ─ the binding of multiple miRNAs to a circRNA and the existence of multiple targets for each miRNA. Analysis of common targets (ACT) was designed to facilitate the identification of potential circRNA targets.

**Results:**

We demonstrated the feasibility of the proposed feature/measurement to assess which genes are more likely to be regulated by circRNAs with given sequences by calculating the level of co-regulation by multiple miRNAs. The web service is made freely available at http://lab-x-omics.nchu.edu.tw/ACT_Server.

**Conclusions:**

ACT allows users to identify potential circRNA-regulated genes and their associated pathways for further investigation.

**Electronic supplementary material:**

The online version of this article (10.1186/s12859-019-2966-3) contains supplementary material, which is available to authorized users.

## Background

Circular RNA (circRNA) is a newly recognized class of single stranded regulatory RNA molecules with ends covalently closed through a backsplice between a downstream splice donor and an upstream splice acceptor. Recent discoveries through sequencing technology and computational analyses have revealed the widespread existence of circRNAs in animal cells and many other organisms [1–3].

CircRNAs contribute to transcriptional activation, post-transcriptional modulation, translation, and protein interactions [4–8]. Among these, the most popularly studied function of circRNA is that of a miRNA sponge that regulates the gene expression network [9–11]. Pioneer studies have made great contributions dissecting and archiving these relationships among miRNAs, circRNAs, and associated pathological phenotypes [12–15]. However, studies investigating the biological functions of circRNAs are largely limited to the scope of a single miRNA linked to a single gene [16–18]. Thus, how to identify a manageable gene list length and to consider its role as a whole for further functional characterization has become a critical task.

In this study, we developed and tested an intuitive concept that genes targeted by more circRNA-associated miRNAs are more likely to be modulated by a given circRNA. We established and provided a web service for the analysis of common targets (ACT) for circRNAs to facilitate the molecular characterization of the biological functions of various circRNAs.

### Implementation

**Fig. 1:**
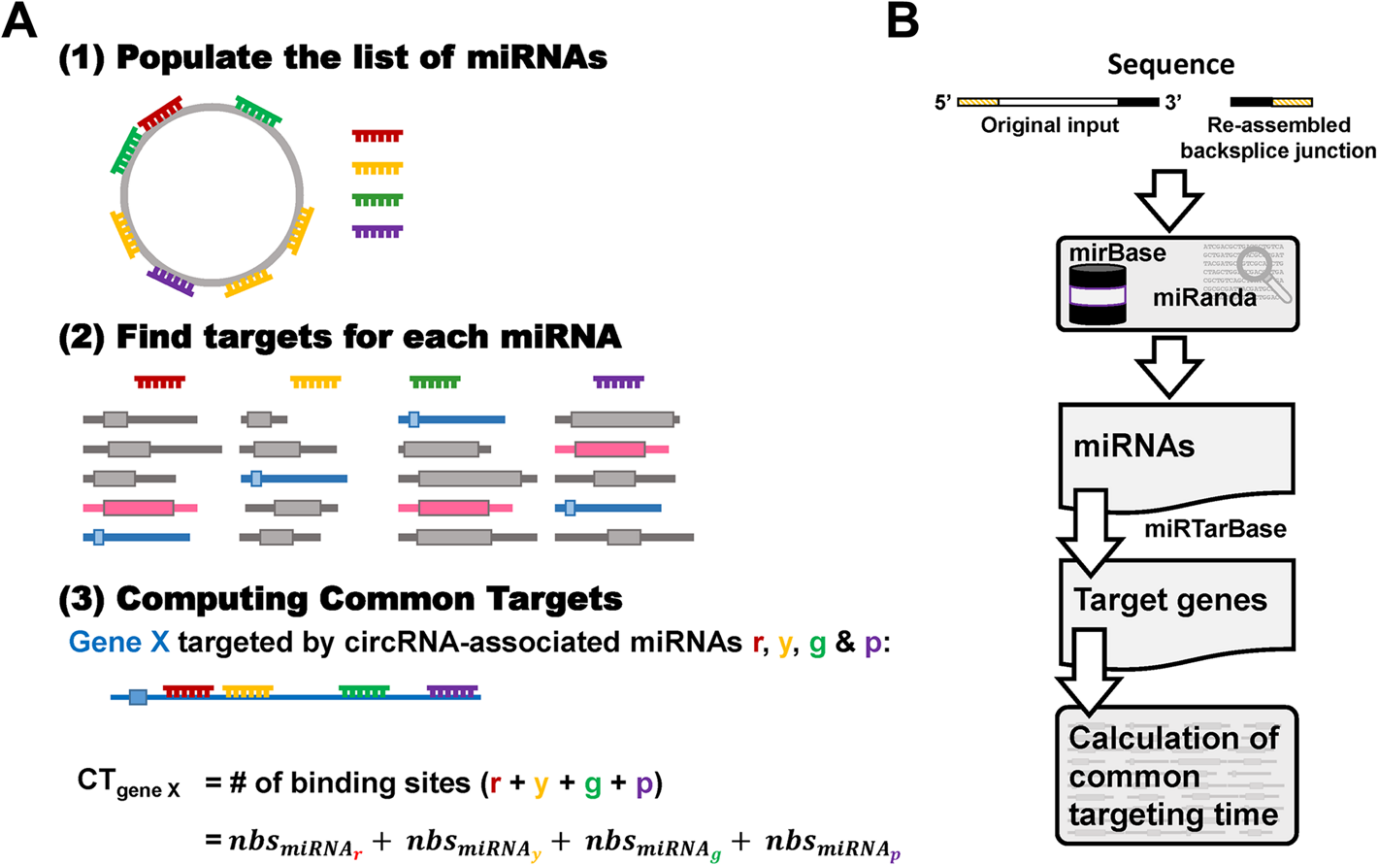
Schematic illustration of ACT. **a** In step 1, miRNAs that bind to the given circRNA were identified by the presence of binding sites. In addition, the number of binding sites (*nbs*) for each miRNA was recorded for further analysis in step 3. In step 2, the targets of each miRNA were identified. It should be noted that some genes (colored in blue and pink) were targets of multiple circRNA-associated miRNAs. In step 3, the *nbs* for miRNAs that bind to the same gene were summed and used for further sorting. **b** The databases and tools integrated in ACT (see the section on implementation)

The circRNA-associated miRNA-gene network typically involves many genes targeted only once by a miRNA (Additional file 1: Figure S1, gray nodes), and thus these genes may be less efficiently regulated by a given circRNA. The central idea of ACT is to identify target genes with high binding numbers for circRNA-associated miRNAs (Additional file 1: Figure S1, blue nodes). To implement this analysis, miRNA-binding sites in circRNAs were first extracted (Fig. 1a - Step 1), followed by the identification of target genes for each circRNA-associated miRNA (Fig. 1 - Step 2). Finally, the targeting number for each gene was calculated (Fig. 1 - Step 3). The flowchart for the sequent processes is summarized in Fig. 1b. The ACT server takes circular RNA sequences in FASTA format. The default miRNA sequences are downloaded from miRBase (Release 22) [19, 20]. First, the user-inputted sequence is extracted from the 5′ and 3′ ends (30 nt) and reverse-joined to produce a backsplice junction. Then the list of miRNAs that potentially bind to the given circRNA is generated by miRanda software (version 3.3a, Aug 2010) [21] using mature miRNA sequences from miRBase. In order to reduce the number of predicted miRNA binding sites, the parameter ‘-strict’ is applied when using miRanda. The position of miRNAs spanning the backsplice junction is calibrated to the beginning of the original inputted sequence, and the number of miRNA binding sites (*nbs*) is recorded for further calculations. A list of the target genes for each miRNA with binding site(s) on the given circRNA is generated using miRTarBase (Release 7.0) [22]. The gene list is then collapsed for unique entries, and for each gene (*g*), the number of targets for circRNA-associated miRNAs (from the last step) is calculated as the sum of *nbs* (see Fig. 1a – Step 3 for example). The genes in the list are ranked by the common targeting time (CT).

$$ Common\ Targeting\ time\ of\ Gene\ g\ \left({CT}_g\right)=\sum \limits_{miRNAs\in g} nbs $$

## Results

### ACT-selected genes are enriched in specific biological pathways

**Fig. 2:**
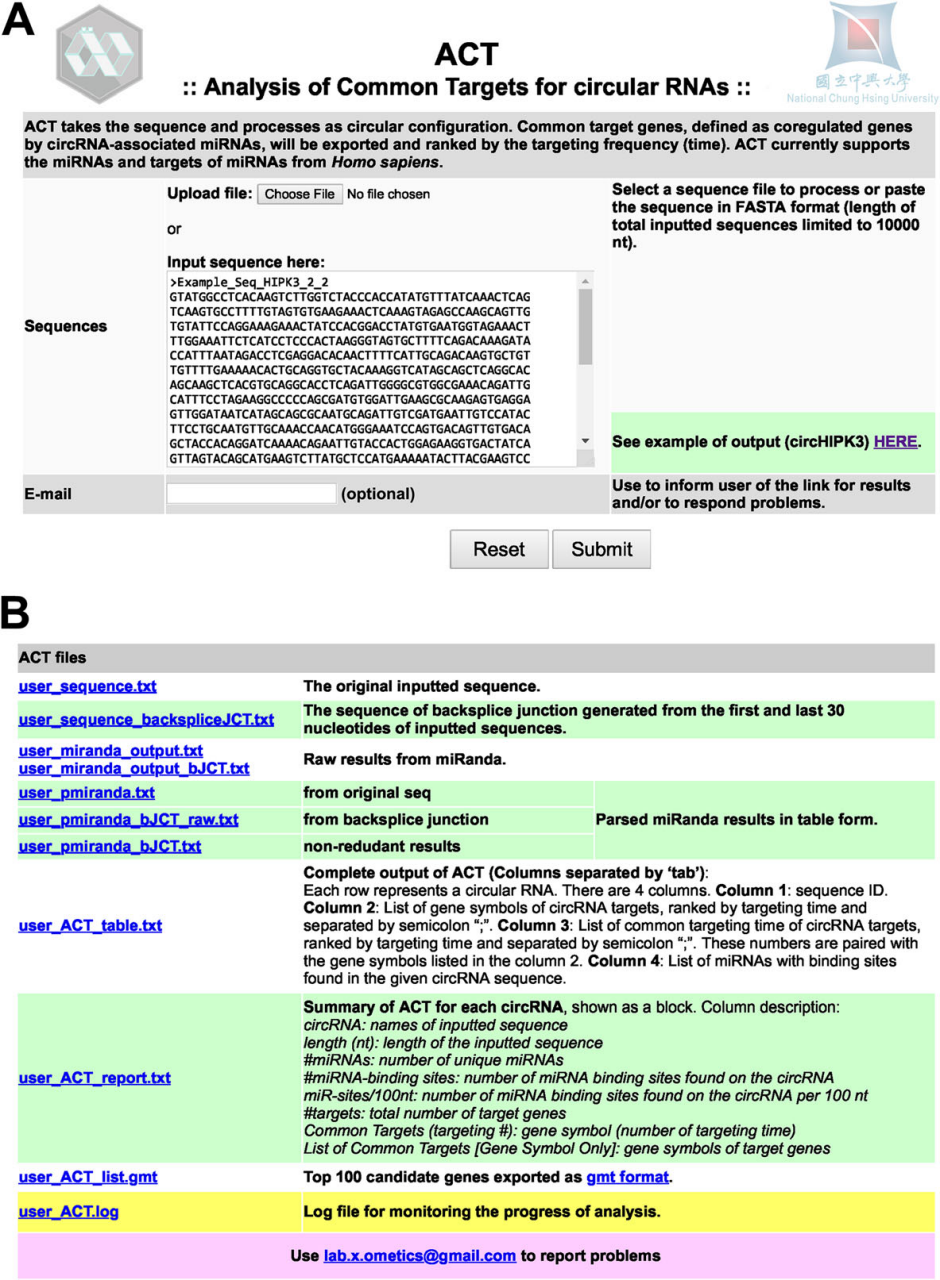
Web interface for ACT. **a** The start page provides a simple and straightforward interface for users to input the necessary information. An example sequence and a link for an example of the analysis are provided (circHIPK3). **b** The analysis example using circHIPK3 is provided with detailed annotation

**Fig. 3:**
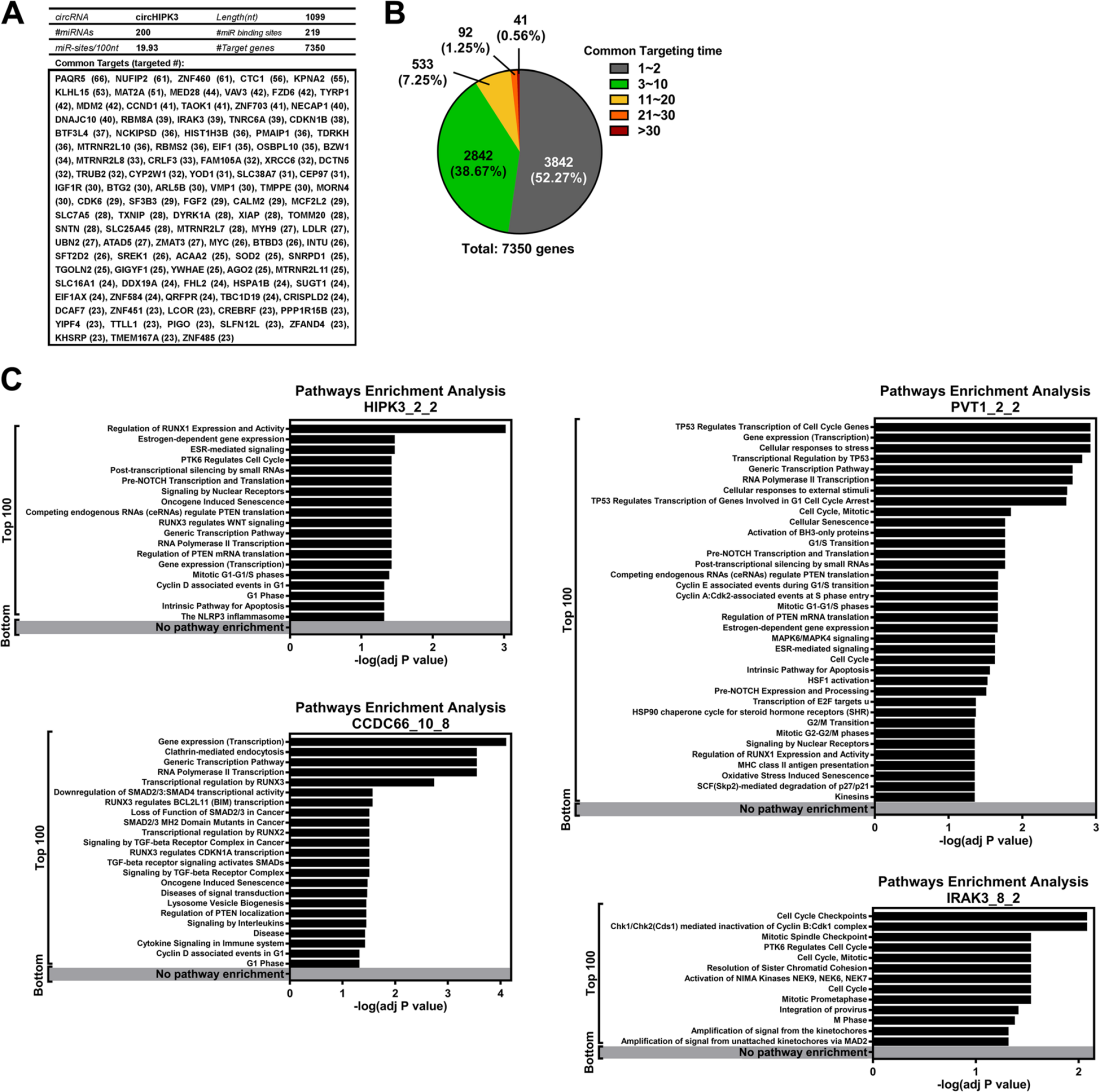
ACT-selected genes identified important biological pathways. **a** The exported ACT results for circHIPK3. The top 100 genes ranked by their common targeting times (parenthesized) are shown at the bottom. **b** The distribution of the common targeting times of circHIPK3-regulated candidate genes is shown as a pie chart. **c** The ACT-prioritized genes (top 100) and low ranked genes (bottom) from circHIPK3, circCCDC66, circPVT1 and circIRAK3 were subjected to pathway enrichment analysis

ACT performs a distinct assessment compared to other metrics that measure the binding energy or pairing score between miRNA and circRNA. Compared to the density of miRNA binding sites, the binding energy and pairing score given by miRanda for these predicted miRNAs on circRNAs, CT for genes provides a more dynamic range to distinguish circRNAs with/without identified miRNA sponge activity and a background dataset (Additional file 1: Figure S2A-D and Table S1). The web interface for ACT is neat and the output files are annotated with detailed information (Fig. 2a and b). CircHIPK3, a previously identified circRNA that targets multiple miRNAs [10], was used as an example (provided for users in the web interface, Fig. 2a). Raw analysis using only the miRNA-target relationship revealed 7350 genes, and approximately half of these genes were targeted by one or two miRNAs (Fig. 3a and b; 3842 out of 7350, 52.27%). ACT is aimed at identifying common targets that are targeted by several circRNA-associated miRNAs. The top 100 genes were exported and are listed in Fig. 3a. A few known circRNAs with and without known sponge activity to multiple miRNAs were used for comparison. Cytoplasmic circRNAs including circHIPK3, circCCDC66, circPVT1 and circIRAK3 [11, 23, 24], previously reported to function as molecular sponges for multiple miRNAs, and nuclear circRNAs from FLI1 and UBR5 genes with distinct molecular functions other than as miRNA sponges in the nuclei [25, 26] were applied to the ACT pipeline. To characterize whether the ACT-predicted circRNA-regulated genes play biological roles, we adapted the concept of co-regulation or the convergence of regulation. We assumed that the circRNA-targeted genes are more likely to be conserved and involved in the same pathways during evolution. The ACT-selected genes were subjected to pathway enrichment analysis. The results of pathway enrichment analysis of the genes selected by ACT from these cytoplasmic circRNAs with miRNA sponge activity demonstrated that these genes tended to be enriched or clustered in the same pathways (Fig. 3c). In sharp contrast, the ACT-selected genes from two nuclear circRNAs (either top- or bottom-ranked ones) showed no pathway enrichment (gene lists provided in Additional file 1: Table S2). The lack of convergence in the regulation of the pathways implied that the molecular functions of these nuclear circRNAs were less likely to be as regulators than as miRNA sponges.

### ACT enables the distinguishment of circRNAs with or without potential miRNA sponge activity

**Fig. 4:**
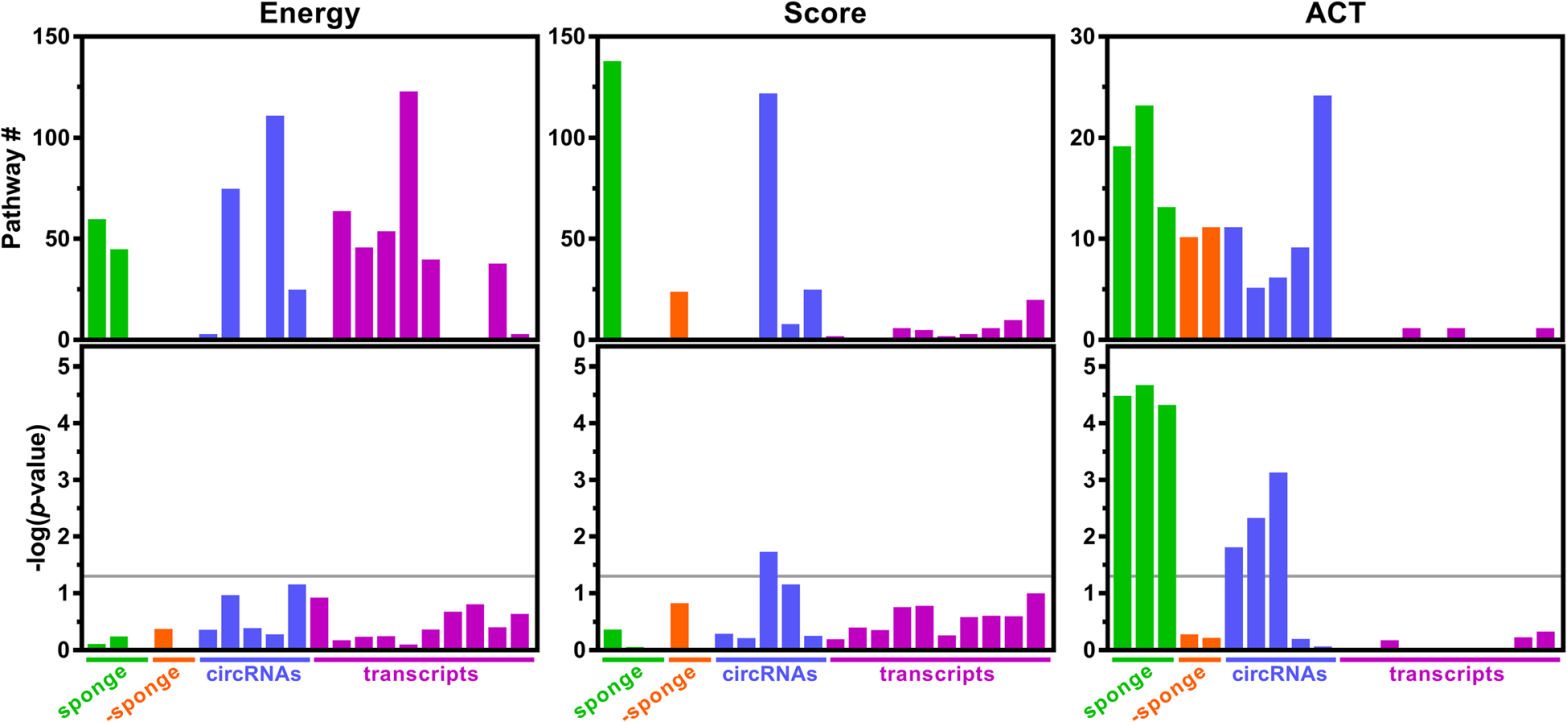
Performance assessment of ACT and alternative miRNA-related metrics The gene lists derived from different ranked metrics (binding energy, pairing score, and ACT) were subjected to pathway enrichment analysis. Each bar represents a circRNA or random transcript according to the label on the x axis. The top row shows the number of pathways while the bottom row shows the *p*-value estimated through a permutation test drawing from the full target gene list of circRNA-associated miRNAs. sponge: circRNAs with known sponge activity to multiple miRNAs. -sponge: nuclear circRNA without known miRNA sponge activity. circRNAs: a group of circRNA with unknown molecular function. Transcripts: random transcript as background dataset

To further elucidate the performance and potential application of ACT, we evaluated the convergence of pathway regulation among different metric-derived gene lists. The target genes of the top 10% of miRNAs according to the pairing score or binding energy in the given circRNA sequence were subjected to pathway enrichment analysis. While enrichment analyses from the gene lists derived from the ranked energy or scores failed to distinguish circRNAs with/without sponge activity from multiple miRNAs (Fig. 4, left and central panels), pathway analyses with ACT-selected gene lists showed significantly more convergent pathways (Fig. 4, right panel). This implied that the genes targeted multiple times by circRNA-associated miRNAs tend to be more biologically significant. ACT is a novel tool to dissect the molecular and cellular functions of circRNAs. Compared to other pioneer databases for annotating circRNA/miRNA interactions (Additional file 1: Table S3) [13, 14, 27], ACT not only provides a list of interacting miRNAs, but also a ranked gene list in a manageable length ready for further functional and/or experimental characterization.

## Conclusion

Taken together, analysis using ACT-selected genes provided a novel and intuitive method to differentiate the molecular and biological functions of circRNAs. Incorporating the concept of co-regulation by multiple circRNA-associated miRNAs provides a straightforward method for assessing the potential targets of circRNAs and will help prioritize the candidates as well as identify major pathways for the functional study of circRNAs.

## Availability and requirements

***Project name*:** ACT

***Project home page*:** http://lab-x-omics.nchu.edu.tw/ ACT_Server/

***Operating system(s)*:** Platform independent (Web-based service)

***Programming language*:** Perl 5 and R 3.4.3

***Other requirements*:** N/A

***License*:** GNU GPL; non-academic user: license needed

## Additional file

Additional file 1: **Figure S1.** A schematic illustration of miRNA-gene interaction. **Figure S2.** Metrics comparison for circRNA-associated miRNAs. **Table S1.** The miRNA-related metrics for circRNA. **Table S2.** Gene lists from ACT for pathway analysis. **Table S3.** The comparison of platforms/tools for circRNA–miRNA–gene network. (PDF 270 kb)

## Data Availability

Web service is made freely available at http://lab-x-omics.nchu.edu.tw/ACT_Server.

## Abbreviations

ACT: Analysis of common targets
circRNA: Circular RNA
CT: Common targeting time
miRNA: MicroRNA
