# Supplementary material for: Analysis of common targets for circular RNAs
Source: BMC Bioinformatics. 2019 Jul 2;20:372. doi: 10.1186/s12859-019-2966-3 (PMC6607602; doi:10.1186/s12859-019-2966-3)
Supplement: Supplementary file 1 — Figure S1. A schematic illustration of miRNA-gene interaction. Figure S2. Metrics comparison for circRNA-associated miRNAs. Table S1. The miRNA-related metrics for circRNA. Table S2. Gene lists from ACT for pathway analysis. Table S3. The comparison of platforms/tools for circRNA–miRNA–gene network. (PDF 270 kb) [file 12859_2019_2966_MOESM1_ESM.pdf]

Supplementary figures

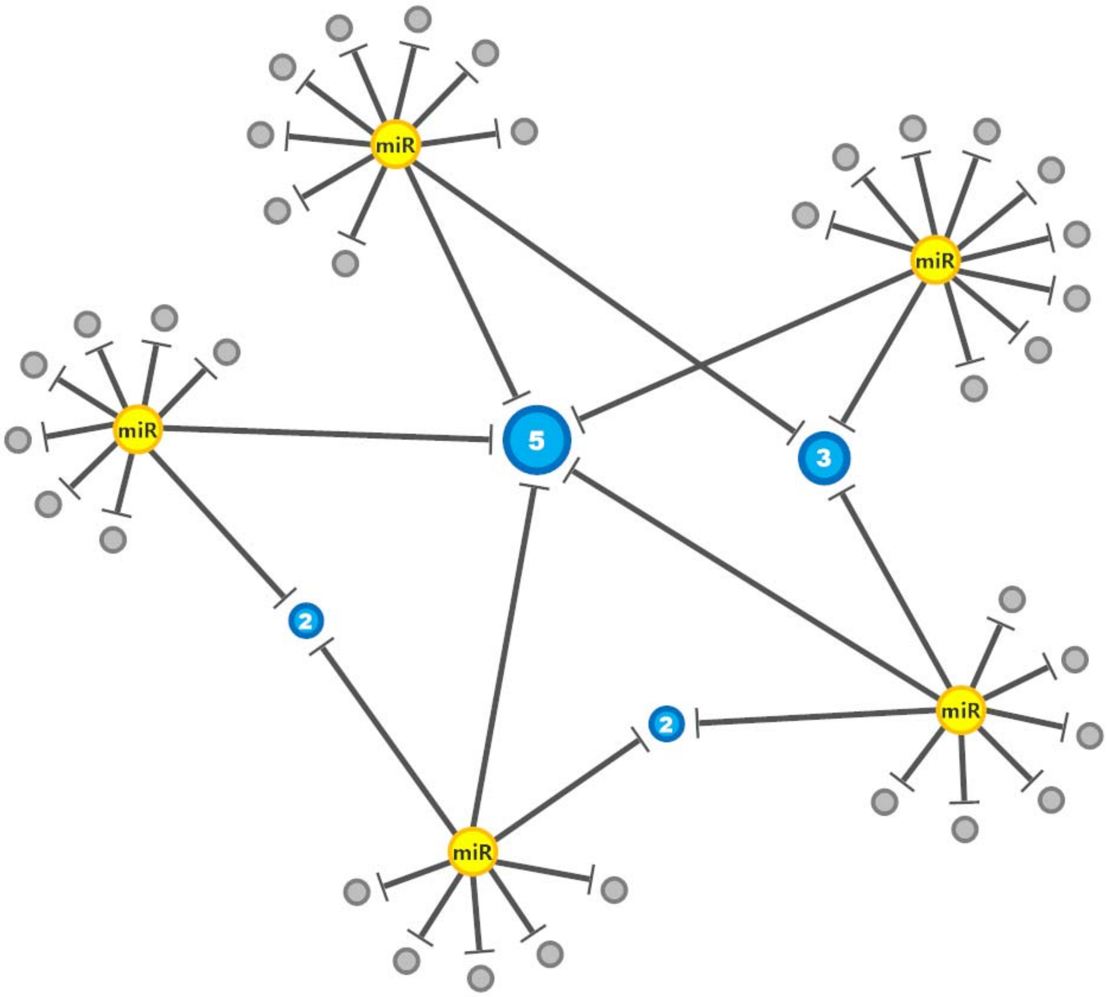

Figure S1 A schematic illustration of miRNA-gene interaction The yellow nodes represent for the circRNA-associated miRNAs. Typically, a single miRNA has dozens of target genes (gray and blue nodes connected by lines to yellow nodes). Most of these target genes from different miRNAs are not frequently overlapped (gray nodes). Certain target genes can be targeted by multiple miRNAs (the blue nodes, the number denotes the targeting time by given miRNAs).

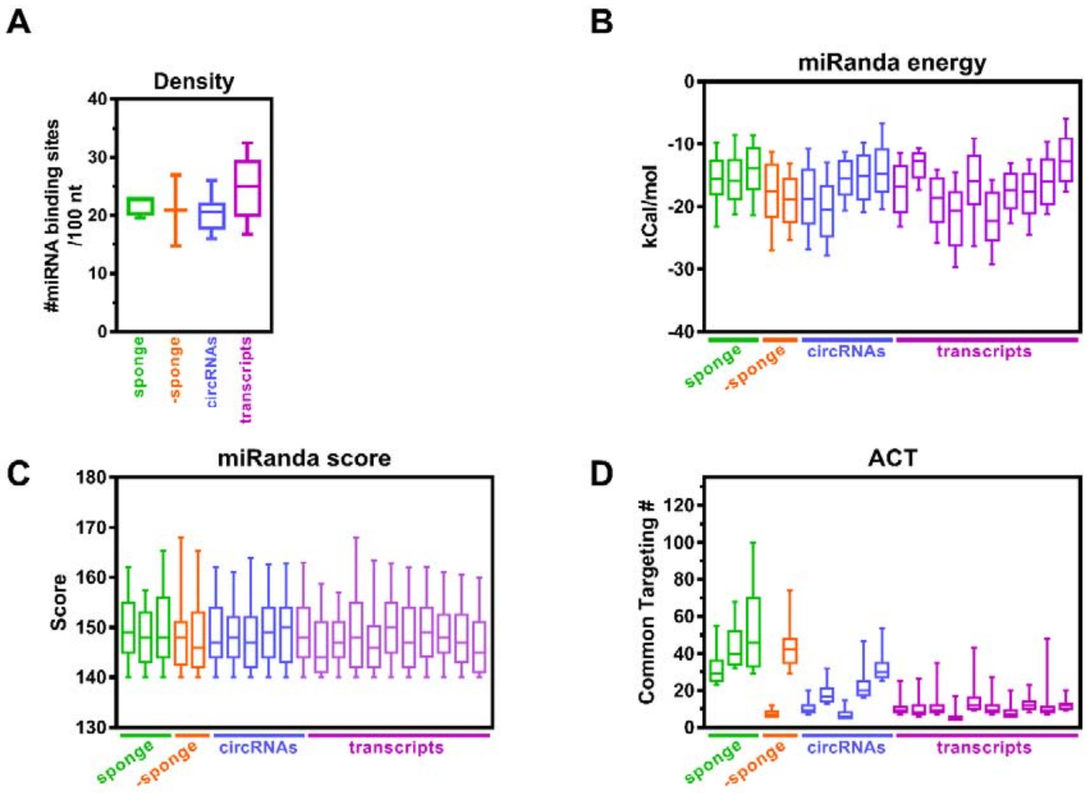

Figure S2 Metrics comparison for circRNA-associated miRNAs (A) The number of miRNA binding sites per 100 nt. **(B)** Binding energy calculated by miRanda. **(C)** Pairing score calculated by miRanda. **(D)** The common target time calculated by ACT. Sponge: circRNAs with known sponge activity to multiple miRNAs. -sponge: nuclear circRNA without known miRNA sponge activity. circRNAs: a group of circRNAs with unknown molecular function. Transcripts: random transcripts as background dataset.

Supplementary tables

Table S1 – The miRNA-related metrics for circRNA

Metric	Subject of measurement	Range scale	Gene # in derived gene list	Gene enrichment in particular pathways
Density of miRNA binding sites	circRNA sequence	~2X	N/A	N/A
Binding energy	miRNA binding site	~3X	$>10^3$	No
Pairing score	miRNA binding site	< 2X	$>10^3$	No
Common Targeting#	gene	>100X	Manageable	Yes

Table S2 Gene lists from ACT for pathway analysis

		Top-ranked		Bottom-ranked	
Cytoplasmic circRNAs	circHIPK3	19†	PAQR5, NUFIP2, ZNF460, CTC1, KPNA2, KLHL15, MAT2A, MED28, VAV3, FZD6, TYRP1, MDM2, CCND1, TAOK1, ZNF703, NECAP1, DNAJC10, RBM8A, IRAK3, TNRC6A, CDKN1B, BTF3L4, NCKIPSD, HIST1H3B, PMAIP1, TDRKH, MTRNR2L10, RBMS2, EIF1, OSBPL10, BZW1, MTRNR2L8, CRLF3, FAM105A, XRCC6, DCTN5, TRUB2, CYP2W1, YOD1, SLC38A7, CEP97, IGF1R, BTG2, ARL5B, VMP1, TMPPE, MORN4, CDK6, SF3B3, FGF2, CALM2, MCF2L2, SLC7A5, TXNIP, DYRK1A, XIAP, TOMM20, SNTN, SLC25A45, MTRNR2L7, MYH9, LDLR, UBN2, ATAD5, ZMAT3, MYC, BTBD3, INTU, SFT2D2, SREK1, ACAA2, SOD2, SNRPD1, TGOLN2, GIGYF1, YWHAE, AGO2, MTRNR2L11, SLC16A1, DDX19A, FHL2, HSPA1B, SUGT1, EIF1AX, ZNF584, QRFPR, TBC1D19, CRISPLD2, DCAF7, ZNF451, LCOR, CREBRF, PPP1R15B, YIPF4, TTLL1, PIGO, SLFN12L, ZFAND4, KHSRP, TMEM167A, ZNF485	0	GLIS3, GCC1, FRRS1L, FOXR2, FBXO9, ENPP5, EIF2B2, DLGAP2, DIP2C, CXCR5, CNPPD1, CHCHD5, CGGBP1, CEP85L, CEP63, CACFD1, C5orf64, C2CD2L, BSN, BMP7, ARSB, ALDH6A1, ABHD10, SCIMP, ARL17B, EPHX2, MLN, MEI1, PLA2G4E, RALGAPA1, SERPINF2, PKHD1L1, OMD, DDO, THEM4, TPGS1, GLUL, DDX53, DBF4, LIAS, SIT1, VAPA, TMEM55A, TMEM161B, SSTR2, SMARCC1, SLC25A25, SKAP2, SH3D19, PSMD1, MRTO4, MRPL51, MFSD6, MAPK10, LGALSL, KCNIP3, KDM7A, GABRA1, CCDC113, C19orf44, REPS2, ZSCAN22, NICN1, PCDHB2, FAM174B, DNAJC3, PRPF6, AGPAT6, GP2, IGF2R, CADPS, CCDC122, AKAP5, TAF1, UBE2G2, FAM120AOS, ULK2, NUDCD2, SRP19, COL8A1, PIWIL1, TRPC3, LAIR1, SNX2, NPHS1, VMAC, C15orf40, PRKX, GLYAT, LRG1, HILPDA, RPL24, CD96, PDE6A, JAM2, ZNF785, SCN3B, PLCXD1, VHLL, RMND1
	circCCDC66	23	RPS27A, LAPTM4A, FBXL5, SOX4, GATA6, PPP1R15B, RAB5B, MIDN, CCND1, LDLR, MYLIP, PDRG1, PTPN4, C11orf30, ZDHHC20, SKIL, CDKN1A, CNOT6L, ANKRD33B, DNAJB9, KPNA2, RAN, NUFIP2, KLHL28, NFAT5, PXK, AKAP11, BTG3, BBX, MARCH6, MAPK1, NABP1, MAP7, MAPRE3, B2M, TMEM127, GIGYF1, TFAM, ARHGAP1, F3, ANKH, TGFBR2, COIL, CNOT4, ZBTB4, BNIP2, SMAD4, FAM129A, FEM1C, LAMTOR1, C3ORF38, GNS, BTF3L4, LAMC1, BCL2L11, HMGB3, U2SURP, NR2C2, EPS15L1, HMGB1, ZC3H12C, PFKP, TXNIP, ZBTB18, FNBP1L, M6PR, ZNF12, ZNF264, ZNF417, ZNFX1, PTEN, NAGK, LIMA1, WASL, SHOC2, PIWIL2, ZNF202, KLHL15, ENPP5, PHTF2, MKNK2, FEM1A, HSPA8, BMP8B, KLHL36, FXYD5, KMT2B, C9orf40, RORA, SEPT2, KIAA1191, KRT10, MDM2, SOD2, DNAJC10, ZFYVE21, MORF4L1, ZNF180, TSG101, PAQR5	0	SCRG1, RGS9BP, REEP2, PRCP, PKHD1, PARVA, OTOG, OLA1, MYO18A, LIMCH1, KDELR1, IPCEF1, HMGCS1, GCNT4, GABRA1, FAT3, ETV3, ESRRG, ERGIC1, DYRK1A, DNAJB5, DIP2C, DIO2, CD200, BHLHE22, ARL3, ALDOA, AEBP2, ABLIM1, NFRKB, CCDC142, ZSCAN22, NICN1, DESI1, PTGIS, CLSTN1, PRPF6, ANGEL2, FAM71F2, ERCC6L2, TOX4, FAM179A, FBXO36, PMPCA, PLA2G4A, ZNF429, MOB3A, PXMP4, SLC10A6, IGF2R, CCDC122, AKAP5, KBTBD6, TAF1, RBBP4, C10orf76, ZNF124, KDELC2, SLU7, MALL, CAPN7, GJD3, FUT2, THAP5, POLR3D, ULK2, TIRAP, SLC35B4, NPR1, RASSF9, SNX1, MAK, NUDCD2, KLHL26, CLEC7A, WWC1, CYP51A1, C9orf69, TMEM91, APTX, KDSR, TNFSF15, ST3GAL2, OGFOD3, CPSF2, PRKX, ITGB3, METTL21A, FITM2, PTRH2, CEP76, LRG1, RHOF, RABAC1, TOP3A, NDUFA7, PRIM2, KIAA0391, JAM2, SCN3B
	circPVT1	36	TXNIP, ANKEF1, CDKN1B, BTBD3, PHF13, TMPPE, DCTN5, CCDC115, SEPT8, VAV3, NAP1L1, MAPK8, HIC2, GIGYF1, ZNF460, YOD1, CHD4, NOL9, BTG2, ZMAT3, MAP3K9, SP4, ARL5C, ZNF354B, INHBA, MIER3, SOCS1, KIF5B, PTP4A1, SRM, FLYWCH2, AKAP11, KPNA4, ZBTB4, MKNK2, KLHL15, MAPKAPK5, ZNF138, EMC7, PPP1R15B, AZIN1, SOX4, CSNK1G1, YWHAE, LARP1, TFDP1, CALM3, TNRC6A, ARID3A, HNRNPA1, PSMD7, MSMO1, MTRNR2L10, PAGR1, SPATA2, KIF23, MSANTD4, DNMT1, SESTD1, RRM2, MED28, TUBB2A, AGO2, IGFBP5, TSPYL1, TMOD3, ARPP19, GATA6, CCNA2, NUFIP2, MAT2A, EML4, HSP90AA1, GOLGA8B, ZNF101, MRPS27, ZFP30, KLF2, CLUAP1, EIF1, LCLAT1, PICALM, POU2F1, DCAF7, ZNF507, KLHL38, TM4SF5, SFT2D2, LMBR1L, AKIRIN1, ADRB1, BARHL1, TXNL1, GREB1, OPHN1, NKTR, ZNF518B, BLOC1S5, RRP36, IL21R	0	DIO2, MPZ, ATP9A, APOOL, FBXL2, TECPR1, WIZ, ZKSCAN3, KCNK5, REPS2, SYNRG, DCUN1D2, SHISA2, NPHP1, BVES, STX4, HOOK3, UHRF1BP1L, CYTIP, MED17, RBM22, PCDHB2, SS18, ATP6V0E1, RAB27A, FOSL2, CBY3, FBXO36, ADK, TRPM6, STIL, MOB3A, PCDHB11, ATP6V0A2, POU2F3, CRX, KIF18B, C22orf29, ZSCAN29, AKAP5, TAF1, ZNF124, RBBP9, RNF24, GTF3C6, ADRBK2, HPSE, VCPIP1, NUDCD2, NDUFA7, SLU7, CDKAL1, INMT, ZNF284, KCMF1, GNE, ULK2, KIF3A, FAM73A, PEX2, PPTC7, SNX1, TTC9C, CLEC7A, HSD17B12, RPL37, COL8A1, RDH10, DHODH, CYP51A1, SLC25A37, PLEKHM3, ZNF107, VMAC, OGFOD3, CPSF2, PCNP, UGGT1, GNB4, GRM6, CEACAM5, DCP1A, SPIC, KLLN, SLC4A4, NECAB1, FITM2, PPAP2B, LCTL, PDCD4, SCUBE3, NMNAT1, MIPOL1, PPP1R3B, ZNF91, C21orf59, SLC25A51, AS3MT, OSBPL2, OXA1L
	circIRAK3	13	ANKEF1, INHBA, PTBP2, SVOP, CDK4, YWHAE, CLIP1, KLHL11, RSBN1, ZNRF2, MTMR6, NPM3, CELSR3, PAFAH1B2, TM9SF3, UBE2D1, PEG10, SMG1, SMIM15, ACTN4, MTHFD2, XRCC6, CAPZA1, LRRC55, FAM3C, ZNF154, HMGN2, FOXN2, AGO2, GDE1, G3BP1, XIAP, SHISA9, CSE1L, SMAD5, SPPL2A, DDIT4, CCNB1, ANP32B, IGF1R, POLR1B, SLC9A4, PTCHD1, ERH, GRPEL2, RPL7L1, SLC5A3, GPR27, WBP4, PLET1, KPNA1, CHEK2, AKAP11, KANSL1, SOX4, SYNCRIP, CDKN1B, YOD1, KLHL28, PARP15, UBE2H, SPRYD4, TRA2B, NHS, ZNF562, KHSRP, PAQR5, XKR9, STRN, SPC25, SIX4, BACH1, GTPBP2, RREB1, ZDHHC21, ETNK1, CBX3, RCC2, HOXA13, NHLRC3, KATNAL1, PDE12, LEPROT, KDM5A, IL6ST, DR1, ZNF207, ZBTB20, MTA3, NUFIP2, TBC1D13, RAB32, PHB2, CYP20A1, HSPA1B, VLDLR, ZNF460, BRIX1, ZWINT, NEK7	0	CISD3, CGGBP1, CEP85L, CDH2, CD28, CCP110, CCDC171, CACNA1E, C1GALT1, BNIP3L, ATP1B4, ARRDC3, ARIH1, AREL1, AQR, AP3M2, AHCY, AFAP1, RABAC1, ZNF841, MPPE1, C8orf82, BMPR1B, UGT2B28, ZNF587B, CCRL2, ADM2, AKAP13, BRSK2, SERPINF2, PKHD1L1, UPF3A, DDO, KCNT2, COA4, LRCH1, MYOZ2, SMYD4, PRX, UGT3A1, TRIM65, LIAS, GALNT5, TNFSF8, ZNF362, SRCIN1, RNF170, RHOBTB3, RBMS2, RALY, POC1A, NT5DC3, LGALSL, KDM7A, HECTD3, ESRRG, ERGIC1, DNAJB5, DIO2, C19orf44, BAHD1, ATOH8, UHRF1BP1L, ERCC6L2, RAB27A, PDE7B, KAT7, IRF1, CADPS, C22orf29, LRRC2, KBTBD6, UBE2G2, THAP1, SLU7, ZNF793, CCBE1, IMP4, PRR23A, C1orf116, ATP5G1, MAK, PIWIL1, C9orf69, OGFOD1, MURC, PDLIM3, CPM, SLC4A4, FBLIM1, HRH4, PDE6A, FAM118A, TTLL12, MANEAL, MAN2A2, LRP10, GMCL1, TOP3A, WDR73

†: the number of enriched pathways

Table S2 Gene lists from ACT for pathway analysis (*continued*)

		Top-ranked	Bottom-ranked
Nuclear circRNAs	circFLI1	0 NCKIPSD, IRAK3, RBM8A, CYP2W1, XRCC6, MCF2L2, SLC25A45, HSPA6, MAT2A, BTF3L4, SNTN, ZNF703, FAM105A, MED28, SLC7A5, TMEM167A, ARL5B, CRLF3, TMEM239, GATA6, SNRPD1, KHSRP, SLC35E2, ZNF451, CEP97, FAM73B, PGAM5, TANGO2, SF3B3, NOA1, DDX19A, MLLT1, ZNF490, RRP7A, IKZF3, TTLL1, OSBPL2, TBC1D19, SLC19A3, FBXW2, ZNF665, CDKAL1, TRUB2, ZNF770, XIAP, TDRKH, TM6SF2, B3GALT5, DNAH17, RBMS2, KIAA0754, ESCO2, PDGFRA, PPEF2, HIST1H2BD, PRRG4, ZFAND4, ZNF860, MYH9, ULBP3, ZSCAN29, MORN4, CRISPLD2, AGO2, ADAR, CLSTN1, SUGT1, ZFP69B, FEM1A, SLC38A7, TIAL1, ACAA2, APOBEC3F, PLAGL2, ZNF584, SLFN12L, ZNF70, DNAJC10, ADAMTS4, C19orf47, PHAX, ONECUT3, MKNK2, GIGYF1, ABI2, ZNF701, SPATA5, PGAM4, MAZ, ZNF573, PLEKHA1, FADS6, LINC00598, LONRF2, HSD17B12, SLC35F6, ZNF394, OPA3, BMP3, METTL14	0 UTP15, UROS, TMEM55A, TMEM168, SLC25A25, SKAP2, SCRG1, RHOBTB3, RGS9BP, REEP2, RCOR1, RALY, RALGPS2, PSMD1, POU2F2, PARVA, NIPAL1, MFSD2A, LUC7L2, KIF26B, ITIH5, FGD4, DPH3, DIP2C, DIO2, CRY2, CIAO1, CDX2, CBX8, C7orf55-LUC7L2, ATOH8, ARPC2, ARL4C, ARL3, CNDP1, STOML1, ISCA2, TECPR1, REPS2, DCUN1D2, GLTP, NPHP1, NICN1, CYTIP, ZNF891, FBXO36, PLEKHS1, CCDC122, C22orf29, KRBA2, LRRC2, KBTBD6, TAF1, SLU7, ZNF333, MALL, KCNN3, ANKRD9, BLOC1S3, FAM120AOS, ULK2, TIRAP, PRR23A, SLC35B4, FAM229B, GLRX2, SNTB2, MAK, NUDCD2, SRP19, TRIP11, WWC1, RDH10, GSTM3, PBOV1, ZNF260, SEC24D, RACGAP1, VSIG1, LAIR1, SNX2, NPHS1, ZNF34, VMAC, NSMCE2, HAUS2, GNB4, ZNF519, EIF2A, METTL21A, ANKRD36, HRH4, XPOT, MAN2A2, GMCL1, HILPDA, RPL24, TLR6, STX2, RMND1
	circUBR5	0 VAV3, HES7, MAPK1, HES4, FEM1A, AP1G1, GATA6, DMPK, SALL1, NECAP1, ADM, MYLK, SREK1, ZNF695, PRRG4, REXO2, SYAP1, SMG1, PLEKHA1, PTMA, UGDH, MTRNR2L7, MTRNR2L11, ERCC1, RBM47, VPS4A, NUFIP2, TNRC6B, PDCL3, SSC5D, HMX2, ABCC5, C15orf38-AP3S2, AP3S2, PRMT7, ZNF460, TCTE1, CDC42EP4, SEMA3E, EHD4, YWHAE, HS3ST1, PITPNM3, MRPS27, ATP5G3, GPR75, LYRM2, RCC1, YTHDC1, ATP8B3, C3, TNRC6C, LINC00598, ZNF286A, PLCXD3, NODAL, MYOCD, MYLK3, MTG1, SYNCRIP, ANKRD52, SH2B3, LRRFIP1, RPL7L1, BTG2, SOWAHC, SOX4, USP42, PEG10, AKAP11, DCTN5, SLC39A9, EIF1AX, ZFP36L1, TMEM239, LYRM4, CALM2, C7orf73, BMP3, ARL5C, PER1, TXNDC16, FGF5, EEF2, RIMS3, PPIG, NAGK, MNX1, KIF23, FGF2, COIL, CDK1, KIF14, RPL27A, MFN2, ZNF431, DMKN, TRIM35, PPIL1, MAGI3	0 HNRNPR, GCC1, FOXR2, FCHSD2, EMB, EIF4E, DNMT3A, CXCR5, CREG2, COX15, CLDN1, CCBE1, C12orf5, C11orf87, BNIP3L, BMPR2, BMPR1A, BCL11B, ARRDC3, ARL5A, ARHGAP29, ARF6, APP, AP3M2, ALDH6A1, AKAP2, ADAM19, FFAR2, SCIMP, ZNF829, ZNF793, MEI1, PGBD4, NFKBID, ICA1L, SEMA5A, LRRC3C, UPF3A, DDO, THEM4, DDX53, BET1L, CXorf56, MYOZ2, C15orf40, JAKMIP3, NIFK, GUF1, L2HGDH, PDE6A, VTA1, UROS, TMTC1, STRBP, SRRM4, SP2, SH3D19, SCRG1, RBMS2, RALY, PCNX, OTOG, NKX2-3, NAV1, ITIH5, GDE1, EIF2AK2, DTWD2, DIO2, CHEK1, ATP9A, AP5M1, ANGEL2, PLA2G4A, KRBA2, ADRBK2, HPSE, ZNF333, MRPL17, ATP5G1, SNTB2, ARL10, SVOP, NUBPL, GSTM3, PLEKHM3, HSPA4L, PDLIM3, TM4SF5, HAUS2, GNB4, FITM2, BPNT1, HRH4, TTLL12, SYK, MIPOL1, RPL24, MSANTD4, OXA1L

Table S3 – The comparison of platforms/tools for circRNA–miRNA–gene network

Interaction presented	Platform: *Description*
**circRNA-miRNA**	**starBase v2.0 and Cancer-Specific CircRNA Database:** *focus on the miRNA binding sites on circRNAs*
**circRNA-miRNA-gene**	**CircNet:** *A network-driven graphical presentation shows the relationship between miRNA target genes and circRNAs with an option to export miRNA list*
	**ACT:** *A web-based tool focuses on the ranked circRNA-regulated genes for pathway analysis*
